# IL-21 Is an Accomplice of PD-L1 in the Induction of PD-1-Dependent Treg Generation in Head and Neck Cancer

**DOI:** 10.3389/fonc.2021.648293

**Published:** 2021-05-05

**Authors:** Yi Zhao, Zhiyu Zhang, Wenbin Lei, Yi Wei, Renqiang Ma, Yihui Wen, Fanqin Wei, Jun Fan, Yang Xu, Lin Chen, Kexing Lyu, Hanqing Lin, Weiping Wen, Wei Sun

**Affiliations:** ^1^ Department of Otorhinolaryngology Head and Neck Surgery, The First Affiliated Hospital of Sun Yat-sen University, Guangzhou, China; ^2^ Guangzhou Key Laboratory of Otorhinolaryngology Head and Neck Surgery, The First Affiliated Hospital of Sun Yat-sen University, Guangzhou, China; ^3^ Department of Biochemistry and Molecular Biology, School of Medicine, Jinan University, Guangzhou, China

**Keywords:** interleukin-21, regulatory T cells, programmed death-ligand 1, tumor microenvironment, head and neck squamous cell carcinoma

## Abstract

Regulatory T cells (Tregs) are immunosuppressive cells involved in antitumor immunity. However, the regulation of Treg generation by inflammation in the tumor microenvironment has not been carefully investigated. Here, we demonstrated that IL-21-polarized inflammation was enriched in the tumor microenvironment in head and neck squamous cell carcinoma (HNSCC) and that IL-21 could promote PD-L1-induced Treg generation in a PD-1-dependent manner. Moreover, generated Tregs showed a greater ability to suppress the proliferation of tumor-associated antigen (TAA)-specific T cells than naturally occurring Tregs. Importantly, an anti-PD-1 antibody could inhibit only Treg expansion induced by clinical tumor explants with high expression of IL-21/PD-L1. In addition, neutralizing IL-21 could enhance the anti-PD-1 antibody-mediated inhibitory effect on Treg expansion. Furthermore, simultaneous high expression of IL-21 and PD-L1 was associated with more Treg infiltrates and predicted reduced overall and disease-free survival in patients with HNSCC. These findings indicate that IL-21 in the tumor microenvironment may promote PD-L1-induced, Treg-mediated immune escape in a PD-1-dependent manner and that an IL-21 neutralization strategy may enhance PD-1 blockade-based antitumor immunotherapy by targeting Treg-mediated immune evasion in patients with high expression of IL-21 and PD-L1.

## Introduction

The interaction between cancer cells and their surrounding microenvironment could modulate the immunoediting process, leading to tumor immune privilege (1–3). In this regard, regulatory T cells (Tregs), which infiltrate a variety of solid tumor microenvironments, are considered a key element in the promotion of immune evasion due to their ability to suppress tumor-specific immune responses and hamper cancer immunotherapy (4–7). In head and neck squamous cell carcinoma (HNSCC), we reported that Treg depletion can repress tumor growth and evoke antitumor immunity and that the accumulation of Tregs predicts poor survival in HNSCC patients (8–10). However, factors that impact Treg generation in the tumor microenvironment are still poorly studied.

We recently found that tumor-associated inflammation (TAI) was positively related to HNSCC tumor-infiltrating Treg generation, which in turn promoted immunosuppression (10, 11). Thus, it would be interesting to further elucidate the potential mechanisms of TAI that are responsible for Treg generation in the tumor microenvironment.

The cytokine Interleukin (IL)-21 is an immune modulator with pleiotropic effects on multiple immune cell type. Driving inflammation by enhancing the generation and functions of cytotoxic T or NK cells is its classical function, importantly, IL-21 also exhibits immunosuppressive properties (12–15). It has been described to mediate the expression of IL-10 by cytotoxic cells and B cells and polarization of tumor-associated macrophage. Furthermore, the effects of IL-21 on immune cells have been reported depending on additional signals (16). The inhibitory checkpoint programmed death-ligand 1(PD-L1) is widely expressed in human malignancies. It interacts through its receptor modulating local immune contexture and serving an inhibitory signal to attenuate anti-tumor immune response. However, little information is currently available regarding interactions between IL-21, PD-L1 and Tregs in local tissue.

In the present study, we identified increased IL-21-polarized inflammation in HNSCC. Moreover, IL-21 could upregulate PD-1 expression on CD4^+^ T cells and boost PD-L1-induced Treg generation through upregulating PD-1 expression. Neutralizing IL-21 could enhance the blockade effect of an anti-PD-1 antibody on Treg generation induced by IL-21^high^/PD-L1^high^ clinical tumor explants. High expression of IL-21/PD-L1 significantly predicted reduced survival in 102 patients with HNSCC. Our findings provide evidence that IL-21-associated inflammation might be modulated within the tumor microenvironment and negatively influence the antitumor immune response.

## Materials and Methods

### Patients and Healthy Donors

One hundred thirty-seven patients diagnosed with laryngeal squamous cell carcinoma at the First Affiliated Hospital of Sun Yat-sen University were recruited for the present study. In detail, immunohistochemistry (IHC) was performed to evaluate the clinical relevance of tumor microenvironment factors in 102 patients, and 35 patients were selected for other *in vitro* studies. None of these 137 selected patients received palliative surgery or neoadjuvant chemo- and/or radiotherapy before sampling. Clinical staging was classified according to the criteria of the seventh edition of the Union for International Cancer Control (UICC). The freshly resected tumor and adjacent non-tumor tissues were kept in cold PBS for downstream analysis. The adjacent non-tumor tissues were confirmed cancer cells infiltration free by pathological examination. Peripheral blood mononuclear cells (PBMCs) were obtained from 14 healthy donors. All samples were collected after receiving informed consent from the patients, and the Ethics Committee of the First Affiliated Hospital of Sun Yat-sen University approved this study (Approval No. 2012-349).

### IHC and Staining Evaluation

Paraffin-embedded, formalin-fixed, 5-μm-thick tissue sections (**Table 1**) were incubated with antibodies against human IL-21 (10 µg/ml, NBP1-02706, Novus), FOXP3 (5 µg/ml, ab20034, Abcam), PD-L1 (1:200, 13684, Cell Signaling Technology), and anti-p16/INK4a (1:250, 10883-1-AP, Proteintech) and then stained using the Dako Envision System (DakoCytomation) according to the manufacturer’s instructions. The procedure for immunohistochemical staining evaluation was described in our previous studies (11). Briefly, for the categorization of samples by IL-21 or PD-L1 expression, specimens with a number of positive cells greater than the median were defined as ‘high’, and those with a number lower than the median were defined as ‘low’. The median level of IL-21^+^ cells was 4.5 cells per field. The expression of PD-L1 was scored semiquantitatively based on the staining intensity and distribution using the immunoreactive score (IRS). The IRS was calculated as staining intensity (SI) × percentage of positive cells (PP) (IRS= SI × PP). The SI was defined as negative (score 0), weak (score 1), moderate (score 2), and strong (score 3). The PP was defined as 0-5% (score 0), 6-25% (score 1), 26-50% (score 2), 51-75% (score 3), and 76-100% (score 4). The cutoff point between low and high PD-L1 expression was 3. P16 was considered positive if there was strong and diffuse nuclear and cytoplasmic staining present in greater than 70% of tumor cells and believed to correlated with Human papilloma virus (HPV). Cells stained with the indicated antibodies were imaged using Zeiss imaging systems (Axio Scan Z1, Carl Zeiss) at 100× and 400× magnification, and at least 5 fields of view per section at 400× magnification were evaluated.

**Table 1:** Clinicopathological characteristics of patients.

Variable		No. cases	%
Gender	Male	95	93.1
	Female	7	6.9
Age (year)	<55	48	47.1
	≥55	54	52.9
Tumor site	Oral cavity	12	11.8
	Nasopharynx	5	4.9
	Larynx	75	73.5
	Hypopharynx	10	9.8
HPV status	p16+	12	11.8
	p16-	90	88.2
Tumor status	T_1-2_	75	73.5
	T_3-4_	27	26.5
Nodal status	N_0_	83	81.3
	N_1-2_	18	18.7
Stage	I+II	70	68.7
	III+IV	32	31.3

### Tissue-Infiltrating Lymphocyte Isolation

Lymphocytes were isolated from tissue as previously described (17). Briefly, fresh surgical specimens (n=17) were minced into small pieces and subsequently digested with Dulbecco’s modified Eagle’s medium (DMEM) containing 5% fetal bovine serum (FBS), 1 mg/ml collagenase I, 0.5 mg/ml collagenase II, 1 mg/ml hyaluronidase, and 100 U/ml DNase (all from Sigma-Aldrich) at 37°C for 20 mins. After being filtered through 70 µm cell strainers, the resulting cell suspensions were processed with density gradient centrifugation using a lymphocyte separation medium (MP Biomedical).

### Flow Cytometry

Single-cell suspensions were stained with antibodies against CD45, CD3, CD4, PD-1, CD25, interferon (IFN)-γ, IL-17, IL-9, IL-4, FOXP3 (eBioscience), and IL-21 (BD Biosciences) according to the manufacturers’ instructions. The dose of each of the above antibodies was 5 µl/test. To detect the cytokine profile, cells were stimulated with Leukocyte Activation Cocktail (2 µl/ml, BD Biosciences) for 5 h before flow cytometric analysis. For intracellular staining, cells were stained with the surface markers listed above and fixed in Fix/Perm Buffer (eBioscience), followed by permeabilization in the presence of antibodies specific for intracellular markers. All the reagents were purchased from BioLegend unless otherwise indicated. The stained cells were acquired on a Cytoflex flow cytometer (Beckman Coulter), and the analysis was performed using FlowJo software (TreeStar).

### Cell Isolation and Culture

CD4^+^ T cells were purified from PBMCs from healthy donors using a CD4^+^ T cell isolation kit (Miltenyi Biotec). Following isolation, the cells were plated at 2×10^5^ cells per well in flat-bottomed 96-well plates in RPMI 1640 medium containing 10% FBS, 2 mM L-glutamine, 0.05 mM 2-mercaptoethanol, and 100 U/ml penicillin and streptomycin. The CD4^+^ T cells were activated using a tetrameric complex of anti-CD3 and anti-CD28 beads (ImmunoCult human T cell activator, STEMCELL). Dendritic cell (DC) preparation was performed as previously described (17), Briefly, CD14^+^ cells isolated from PBMCs were cultured at a density of 1 × 10^5^ cells per well in RPMI 1640 medium supplemented with GM-CSF (50 ng/ml, 300-03, PeproTech) and recombinant human IL-4 (20 ng/ml, 200-04, PeproTech) for 6 days.

#### Preparation of Tumor-Associated Antigen (TAA)-Specific T Cells

Autologous TAA-specific T cells were prepared using antigen-loaded DCs as we previously described (17). Briefly, soluble SNU899 cell line lysate antigens prepared by four freeze-thaw cycles (-140°C/42°C/60°C) were added to DC cultures at day 6 at a ratio of 3:1 (SNU899 cells: DCs). Lipopolysaccharide (LPS, 1 µg/ml, Sigma-Aldrich) was added to induce DC maturation. The maturation of the DCs was determined by measuring the expression of HLA-DR, CD80, CD86, and CD83 by flow cytometry. Antigen-loaded DCs were added to autologous T cells as stimulators at a ratio of 1:20.

#### Treg Generation Asay

CD4^+^ T cells were activated using a tetrameric complex of anti-CD3/CD28 beads (ImmunoCult human T cell activator, STEMCELL) with or without recombinant human IL-21 (25 ng/ml, R&D Systems). Recombinant human PD-L1 (rhPD-L1, R&D Systems) was incubated at a concentration of 100 ng/ml in 96-well plates overnight in the presence of a goat anti-human IgG Fc antibody for dimerization and plate immobilization. Tregs were generated by stimulating CD4^+^ T cells with anti-CD3/CD28 beads for 6 days with or without the presence of immobilized PD-L1 or IL-21. To study Treg generation, which may be influenced by the tumor microenvironment, tumor slices from clinical specimens (n= 10) were laid on the bottom of the wells of a 96-well plate and cocultured with CD4^+^ T cells isolated from PBMCs in the presence of anti-CD3/CD28 beads. For neutralization studies, recombinant human IL-21 R Fc Chimera (10 µg/ml, R&D Systems) and an anti-human PD-1 antibody (10 µg/ml, R&D Systems) were added at the start of the coculture. Cells were harvested at the indicated time and then analyzed with a Cytoflex flow cytometer.

#### Treg-Mediated Suppression of TAA-Specific T Cells

Using the Regulatory T Cell Isolation Kit (Miltenyi Biotec), generated CD4^+^CD25^+^CD127^low^ Tregs were isolated from CD4^+^ T cells stimulated with anti-CD3/CD28 beads and seeded together with autologous TAA-specific T cells labeled with carboxyfluorescein diacetate succinimidyl ester (CFSE, 1 μM, eBioscience) at a ratio of 1:2 with a total of 30,000 cells per well.

### Statistical Analysis

All statistical analyses were performed using IBM SPSS software version 22.0 (IBM Corporation). The Kaplan-Meier method was used to evaluate survival distributions, and differences between groups were assessed by the log-rank test. The correlation between variables was evaluated using Spearman’s correlation test. Two-sided Student’s t tests and ANOVA were used to analyze data from IHC and flow cytometry experiments, and the Bonferroni adjustment for multiple comparisons was used for between-group comparisons. Data are presented as the mean ± SD. P-values <0.05 were considered statistically significant.

## Results

### IL-21-Polarized Inflammation Is Enriched in the HNSCC Tumor Microenvironment

**Figure 1:**
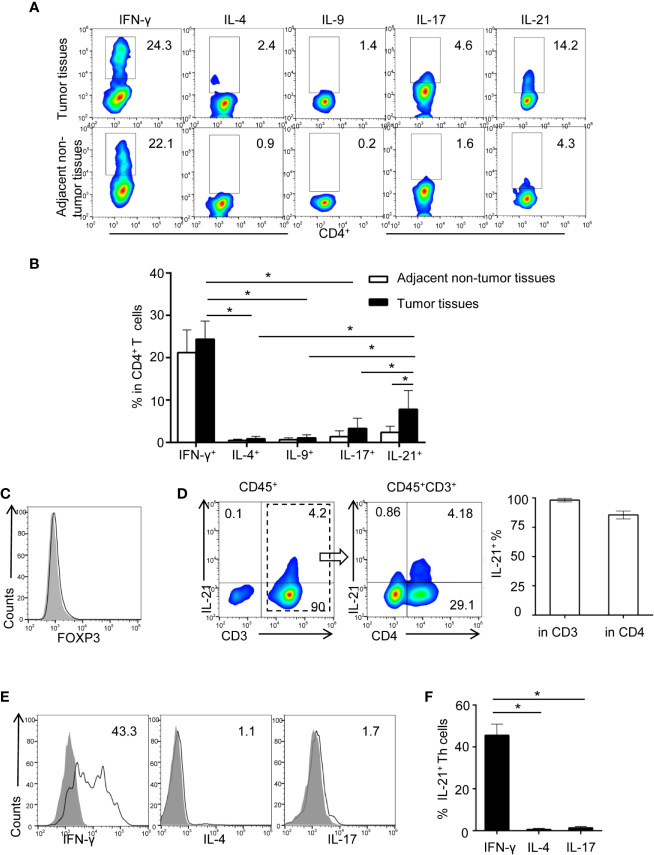
Identification of increasing tumor-infiltrating IL-21 producing Th cells in HNSCC. **(A)** Representative flow cytometric plots of Th subsets isolated from tumor and adjacent non-tumor tissues. **(B)** Quantification of proportions of Th subsets (mean ± SD, n = 9, *P < 0.05 by Student’s t test). **(C, D)** Flow cytometric analysis of IL-21 frequency in gated CD45^+^ cells and CD45^+^CD3^+^ cells in tumor infiltrating lymphocytes. **(E, F)** Expression profile of IFN-γ, IL-4, and IL-17 in IL-21^+^ Th cells from HNSCC tumor tissues, n = 5. Gating strategy was provided in **Supplementary Figure 1**.

We previously showed evidence that tumor-associated inflammation (TAI) was enriched in the HNSCC tumor environment (10, 17). However, the type of tumor environment inflammation that dominates the regulation of tumor immunity in HNSCC is unknown. In the present study, HNSCC tumor-infiltrating lymphocytes (TILs), which secrete typical cytokines associated with Th1-, Th2-, Th9-, Th17-, and Th21-polarized inflammation (including IFN-γ, IL-4, IL-9, IL-17, and IL-21), were identified using flow cytometric analysis. Our results showed that the frequencies of IFN-γ^+^CD4^+^ (26.2 ± 4.2%) and IL-21^+^CD4^+^ T cells (8.03 ± 4.37%) in tumor tissue were higher than those of IL-4^+^CD4^+^ (0.8 ± 0.5%, P< 0.01 and P< 0.01, respectively), IL-9^+^CD4^+^ (1.2 ± 0.7%, P< 0.01 and P< 0.01, respectively), and IL-17^+^CD4^+^ T cells (3.2 ± 2.6%, P< 0.01 and P< 0.01, respectively), which indicated that IFN-γ and IL-21 may be the dominant secreted cytokines of tumor-infiltrating Th cells (**Figures 1A, B**, **Supplementary Figure 1**).

We next compared the differences in the IFN-γ^+^CD4^+^ and IL-21^+^CD4^+^ T cell frequencies between tumor and adjacent non-tumor tissues. Notably, our results showed that despite the high frequency of IFN-γ^+^CD4^+^ T cells in the tumor tissues, the frequency of IFN-γ^+^CD4^+^ T cells was not considerably different between the tumor and adjacent non-tumor tissues (26.2 ± 4.2% *vs.* 22.8 ± 4.8%, respectively, P > 0.05) (**Figures 1A, B**). However, the frequency of IL-21^+^CD4^+^ T cells was significantly increased in the tumor tissues compared with the paired adjacent non-tumor tissues (8.03 ± 4.37% *vs.* 2.4 ± 1.4%, respectively, P < 0.01) (**Figures 1A, B**). Moreover, the IL-21-producing T cells in the tumor tissues were FOXP3 negative, and the majority of these cells (84.5 ± 3.3%) were CD3 and CD4 positive (**Figures 1C, D**). Finally, we found that approximately half of the IL-21^+^CD4^+^ T cells in the tumor tissues were IFN-γ positive (45.6 ± 5.2%) and that IL-21^+^CD4^+^ T cells were rarely IL-4 (0.8 ± 0.3%) or IL-17 (1.6 ± 0.5%) positive (**Figures 1E, F**).

### IL-21^+^ Cells Predict Poor Survival in Patients With HNSCC

**Figure 2:**
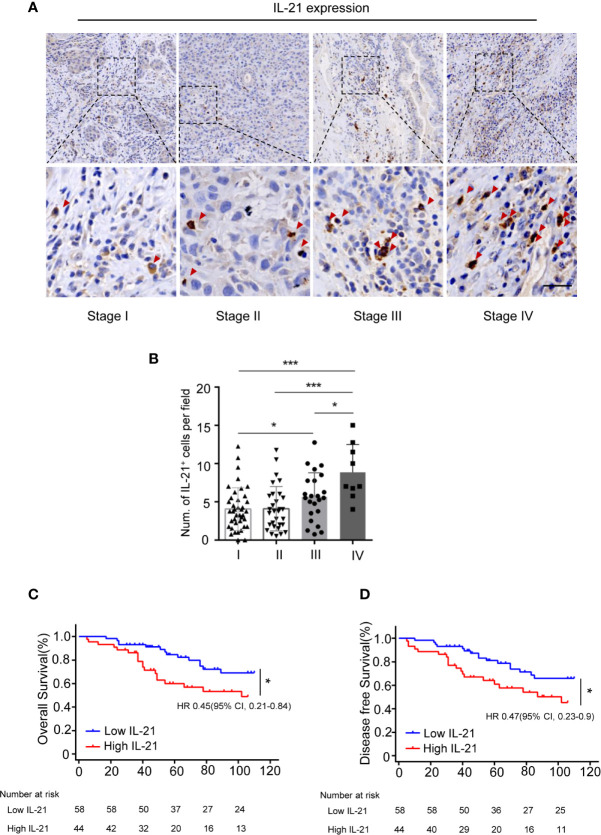
Accumulation of IL-21^+^ cells are associated with poor prognosis of patients with HNSCC. **(A)** Representative images showing staining of IL-21^+^ cells within the tumor stroma. Positive cells are stained brown. (Red arrowheads, Scale bar, 50μm). Bottom panels (400×)are magnified images of the boxed area in the corresponding upper panel (100×). **(B)** IL-21^+^ cell infiltration in patients with each of the stage I, II, III and IV (n=102, *P < 0.05, ***P < 0.001, by One way ANOVA.). **(C, D)** Kaplan-Meier survival curve of HNSCC patients with low and high numbers of tumor infiltrating IL-21^+^ cells, compared by the log-rank test. Patients with high IL-21^+^ cells levels had significantly poorer overall survival **(C)** and disease-free survival **(D)** compared with individuals with low.

We subsequently evaluated the clinical relevance of the increased frequency of tumor-infiltrating IL-21-producing cells in 102 HNSCC patients using IHC. Our results showed that IL-21^+^ cells accumulated in the tumor stroma but not in the tumor nest (**Figure 2A**). The levels of IL-21^+^ cell infiltration in patients with stage IV disease were higher than those with stage I, II or III disease (8.9 ± 3.6 *vs.* 4.0 ± 2.7, P < 0.001, *vs.* 4.1 ± 2.9, P < 0.001, *vs.* 5.6 ± 3.1, P < 0.05, counts per field, respectively), Additionally, those with stage III disease was higher than stage I (5.6 ± 3.1 *vs.* 4.0 ± 2.7, P < 0.05, counts per field) (**Figures 2A, B**).

To determine whether IL-21^+^ cell infiltration in HNSCC correlates with disease prognosis, one hundred and two HNSCC patients were divided into two groups according to the median value of their IL-21^+^ cell density. The patients with a high level of infiltrating IL-21^+^ cells had worse overall and disease-free survival than the patients with a low level (**Figures 2C, D** and **Table 2**). Cox regression analysis revealed that the IL-21^+^ counts and TNM stage could be independent predictive factors for overall survival in the HNSCC patients (**Table 3**). Taken together, our results identified that the accumulation of IL-21^+^ cells was associated with disease progression and poor prognosis in patients with HNSCC.

**Table 2:** Relationships between tumor stromal IL-21^+^ cells and clinical variables.

Variable		stromal IL-21+ cells		P value
		Low (cases)	High (cases)	
Gender	Male	54	41	0.073
	Female	1	6	
Age, years	<55	25	23	0.879
	≥55	30	24	
Tumor site	Oral cavity	7	5	0.707
	Nasopharynx	4	1	
	Larynx	41	34	
	Hypopharynx	5	5	
HPV status	p16+	8	4	0.346
	p16-	47	43	
Tumor status	T_1-2_	46	29	**0.023**
	T_3-4_	9	18	
Nodal status	Negative	50	33	**0.044**
	Positive	6	13	
Stage	I+II	44	26	**0.013**
	III+IV	11	21	

The bold value indicates statistical significance.

**Table 3:** Univariate and multivariate analyses of factors associated with survival and recurrence.

	OS				DFS			
	Univariate		Multivariate		Univariate		Multivariate	
	P	HR	95%CI	P	P	HR	95%CI	P
Gender(Male)	0.366				0.532			
Age(≥55)	0.431				0.739			
Tumor site(Larynx)	0.541				0.675			
HPV status(p16+)	0.673				0.714			
Tumor status (T_3_-T_4_)	**0.041**	1.43	0.73-2.32	0.837	0.065			
Nodal status (Positive)	**0.012**	1.52	1.18-2.76	0.203	**0.026**	1.23	0.76-1.91	0.253
Stage (III-IV)	**0.003**	2.03	1.32-3.28	**0.023**	**0.007**	1.17	0.62-1.89	**0.035**
IL-21^high^	**0.013**	1.83	1.06-2.72	**0.007**	**0.032**	1.28	0.87-1.50	0.112
IL-21^high^PD-L1^high^	**<0.001**	2.71	1.24-4.85	**0.004**	**<0.001**	1.62	1.02-2.46	**0.019**

The bold value indicates statistical significance.

### Abundance of IL-21^+^ Cells Is Positively Correlated With Treg Infiltration in HNSCC

**Figure 3:**
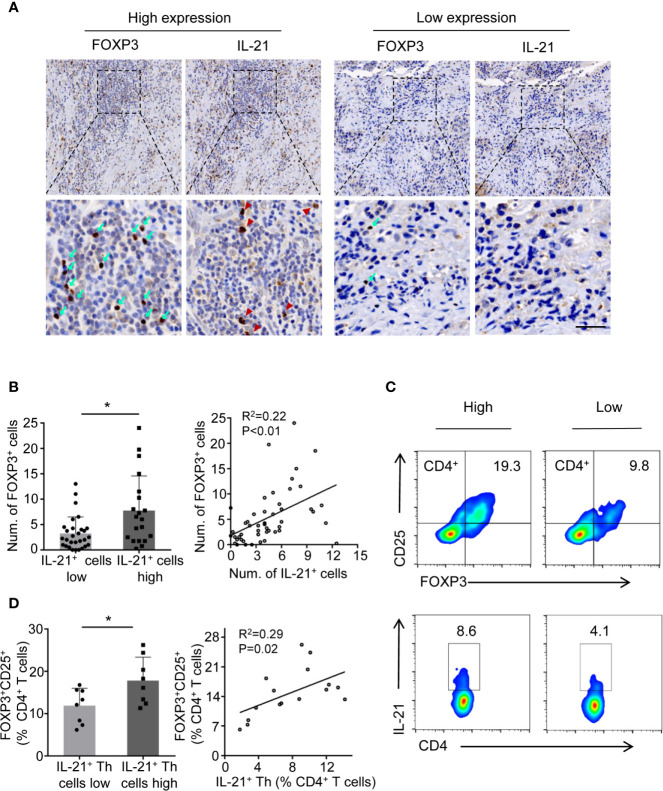
IL-21^+^ Th cells accumulation is positively correlated with Treg infiltration in HNSCC tissues. **(A)** High and low expression of FOXP3 (cyan arrows) and IL-21 (red arrowheads) positive cells at the same area of stroma. Scale bar, 50μm, magnification, bottom panels 400×, upper panel 100×. **(B)** Correlation between FOXP3^+^ and IL-21^+^ cells using immunohistochemical analysis. n=49. **(C)** Representative flow cytometric plots of tumor infiltrating FOXP3^+^ CD25^+^CD4^+^ and IL-21^+^CD4^+^ T cells. **(D)** Correlation between FOXP3^+^ and IL-21^+^ cells using flow cytometric analysis. n=16. In immunohistochemical analysis, the median level of IL-21^+^ cells was 4.5 cells per filed. In flow cytometric analysis, the median percent of IL-21^+^ Th in CD4^+^ T cells was 7.75%. (*P < 0.05 by Student’s t-test).

Considering that Tregs are known to suppress antitumor immunity and that this suppression results in disease progression, we next determined whether the level of IL-21^+^ cells was related to Treg infiltration. We first examined the prevalence of tumor-infiltrating Tregs and IL-21^+^ cells using immunohistochemical staining for FOXP3 and IL-21. The results showed that FOXP3^+^ and IL-21^+^ cells collocated in the same area in the tumor stroma (**Figure 3A**) and that the increased density of the IL-21^+^ cells was positively correlated with that of the FOXP3^+^ cells (**Figure 3B**). Next, flow cytometric analysis showed that the frequency of tumor-infiltrating IL-21^+^CD4^+^ T cells positively correlated with that of FOXP3^+^CD25^+^CD4^+^ T cells (**Figures 3C, D**), which supported our immunohistochemical results. We further assessed IL-21R expression on Treg cells and conventional T cells (CD45^+^CD3^+^CD4^+^CD8^-^CD25^-^FOXP3^-^), showing that Tregs have remarkably more IL-21R expression (**Supplementary Figure 2**). These results implied that IL-21^+^ cells may be involved in Treg infiltration in the HNSCC tumor microenvironment.

### IL-21 Promotes PD-L1-Induced Treg Generation in a PD-1-Dependent Manner

**Figure 4:**
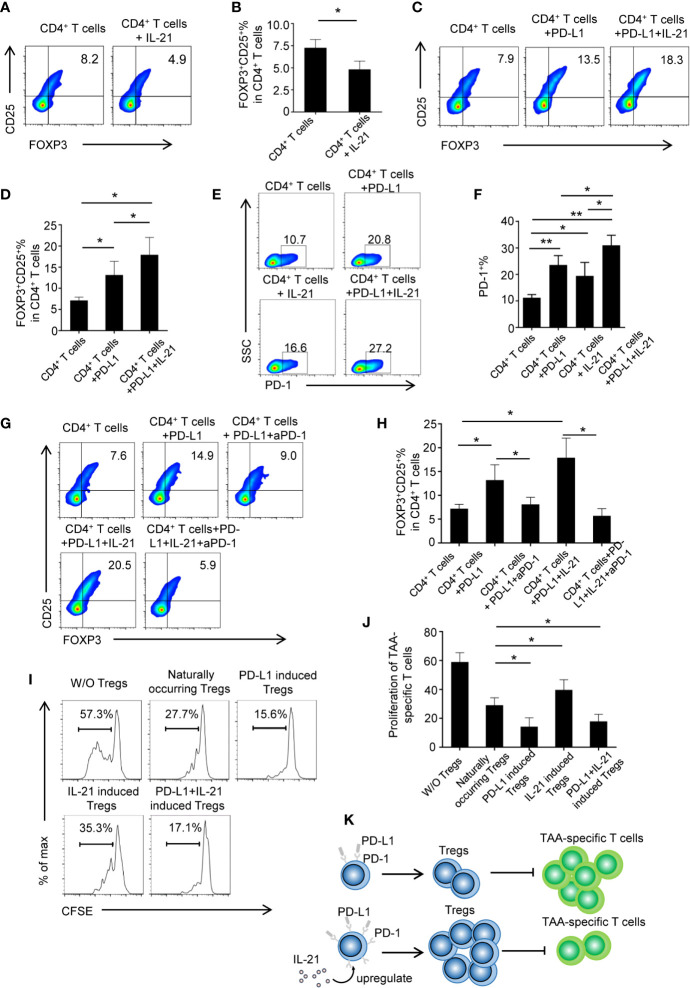
IL-21 contribute to PD-L1 induced Tregs generation in PD-1 dependent manner. **(A, B)** CD4^+^ T cells from human peripheral blood were stimulated with anti CD3/28 antibody coated beads with or without IL-21. The percentage of FOXP3^+^CD25^+^ Tregs were quantitated and shown. (n = 5, *P < 0.05, by Student’s t-tests.) **(C, D)** CD4+ T cells were treated with or without PD-L1 or/and IL-21. Percentage of FOXP3^+^CD25^+^ Tregs was shown (n = 5, *P < 0.05, by Student’s t-tests). **(E, F)** Proportion of PD-1 expressing CD4^+^ T cells in the presence or absence of IL-21 or PD-L1 (n = 4, *P < 0.05, **P < 0.01 by one way ANOVA). **(G, H)** CD4+ T cells were treated with or without PD-L1 or/and IL-21 in the presence or absence of PD-1 neutralization antibody. Percentage of FOXP3^+^CD25^+^ Tregs was shown (n = 5, *P < 0.05, by Student’s t-tests) **(I, J)** Induced Tregs were cocultured with CFSE labeled TAA-T responder cells at ratios of 1:2 and in the presence of anti-CD3/anti-CD28 antibodies. Proliferation of responder cells were assessed after 72h by flow cytometry. Plots are representative of five separate experiments, *P < 0.05, compared with naturally occurring control Tregs by Student’s t-tests. **(K)** Schematic figure illustrating that IL-21 promoted PD-L1-induced Treg generation in a PD-1-dependent manner, IL-21 and PD-L1-induced Tregs inhibited TAA-specific T cell proliferation at a greater degree than naturally occurring Tregs.

To determine whether tumor-infiltrating IL-21^+^ cells contribute to Treg generation, recombinant human IL-21 was added to cultured CD4^+^ T cells. However, the unexpected results showed that the frequency of Tregs markedly declined in the CD4^+^ T cells + IL-21 group compared with the CD4^+^ T cells alone group (4.8 ± 0.8% *vs.* 7.2 ± 0.9%, respectively, P < 0.05) (**Figures 4A, B**), and this result was inconsistent with our clinical data. We speculated that IL-21 may have the effect of antagonizing Treg when treated alone, which was supported by literature demonstrating the pathogenic role of IL-21 in human inflammatory diseases, and may act in concert with other immunosuppressive factors in Treg generation in the tumor microenvironment. As the PD-L1/PD-1 pathway has been recognized as a molecular checkpoint in immune evasion in various cancers (18–22), we next examined the effect of IL-21 on Treg generation from CD4^+^ T cells in the presence of PD-L1. Excitingly, our results showed that the frequency of Tregs was not only higher in the CD4^+^ T cells + PD-L1 group than in the CD4^+^ T cells alone group (13.2 ± 3.2% *vs.* 7.2 ± 0.9%, respectively, P < 0.05) but also higher in the CD4^+^ T cells + PD-L1 + IL-21 group than in the CD4^+^ T cells + PD-L1 group (17.9 ± 4.1% *vs.* 13.2 ± 3.2%, respectively, P < 0.05) (**Figures 4C, D**), which meant that IL-21 could enhance PD-L1-induced Treg generation. We next examined whether this Treg generation was PD-1 dependent, and the results showed that compared with PD-L1 (23.5 ± 3.5%, P < 0.05) or IL-21 alone (19.4 ± 5.1%, P < 0.05), the combined use of PD-L1 and IL-21 (32.0 ± 4.4%) markedly upregulated PD-1 expression on CD4^+^ T cells (**Figures 4E, F**), suggesting a synergistic effect of IL-21 and PD-L1 on PD-1 induction. Most importantly, blocking PD-1 with a neutralizing antibody abrogated the effects of IL-21 and/or PD-L1 on Treg generation (*vs.* IL-21+PD-L1: 5.7 ± 1.5% *vs.* 17.9 ± 4.1%, P < 0.05; *vs.* PD-L1: 8.1 ± 1.5% *vs.* 13.2 ± 3.2%, P < 0.05) (**Figures 4G, H**), which indicated that IL-21 and PD-L1-induced Treg generation was PD-1 dependent.

### PD-L1 and IL-21-Induced Tregs Show a Stronger Ability to Suppress the Proliferation of TAA-Specific T Cells Than Naturally Occurring Tregs

For the analysis of the functionality of the generated Tregs, Tregs were isolated from different groups and cocultured with CFSE-labeled TAA-specific T cells. Our results showed that the generated Tregs from the CD4^+^ T cells + PD-L1 + IL-21 group (17.8 ± 4.9%, P<0.05) or the CD4^+^ T cells + PD-L1 group (14.3 ± 6.1%, P<0.05) had stronger suppressive effects on TAA-specific T cell proliferation than those from the CD4^+^ T cells alone group (29.0 ± 5.2%), whereas compared with those from the CD4^+^ T cells alone group, the Tregs isolated from the CD4^+^ T cells + IL-21 group showed an impaired suppressive ability (39.7 ± 7.0% *vs.* 29.0 ± 5.2%, respectively, P<0.05) (**Figures 4I, J**). In addition, TAA-specific CD8^+^ T cells were assessed as well (**Supplementary Figure 3**). It revealed that PD-L1 and IL-21-induced Tregs show expected suppressive effects as on TAA-specific CD8^+^ T cells. Taken together, schematic figure (**Figure 4K**) illustrates that IL-21 promoted PD-L1-induced Treg generation in a PD-1-dependent manner, and IL-21 and PD-L1-induced Tregs could inhibited TAA-specific T cell proliferation at a greater degree than naturally occurring Tregs.

### Neutralizing IL-21 Enhances the Blockade Effect of an Anti-PD-1 Antibody on the Treg Generation Induced by IL-21^high^/PD-L1^high^ Tumor Explants but Not on that Induced by IL-21^low^/PD-L1^low^ Tumor Explants

**Figure 5:**
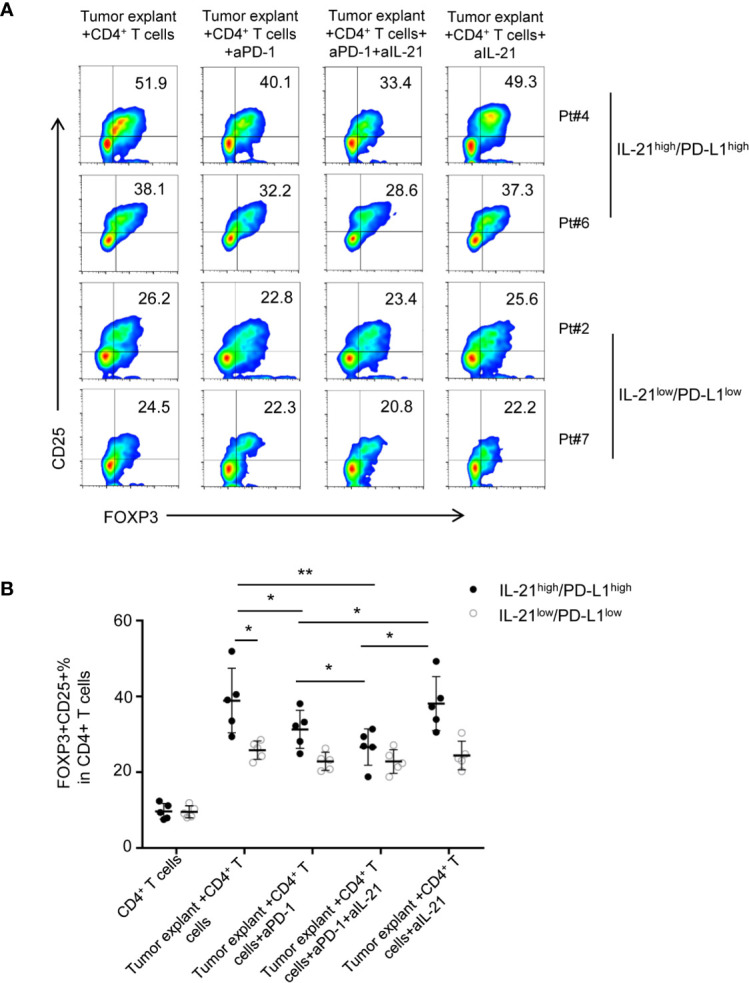
Neutralizing IL-21 enhances the blocked effect of PD-1 antibody on Treg generation induced by IL-21^high^/PD-L1^high^ tumor explants. **(A)** Tumor explants from 10 patients with HNSCC were divided into 2 groups according to the cutoff points of PD-L1 and IL-21. Effect of HNSCC tumor explant on Treg induction was assessed by culturing tumor explants and CD4^+^ T cells isolated from peripheral blood in the presence or absence of IL-21 and/or PD-1 neutralization antibody. **(B)** Quantification of percentage of FOXP3^+^CD25^+^ Tregs was shown (n = 5, *P < 0.05, **P < 0.01, by Student’s t-test).

To test whether the HNSCC tumor environment is capable of potentiating Treg generation through IL-21 and PD-L1, we cocultured CD4^+^ T cells with tumor explants from HNSCC patients with or without IL-21 and/or an anti-PD-1 neutralizing antibody. Accordingly, tumor explants from 10 patients with HNSCC were divided evenly into 2 groups according to the cutoff points for PD-L1 and IL-21 described in the methods section. There were 5 samples with high expression of both IL-21 and PD-L1 (IL-21^high^/PD-L1^high^) and 5 samples with low expression of both IL-21 and PD-L1 (IL-21^low^/PD-L1^low^). Our results showed that the frequency of Tregs in the group of IL-21^high^/PD-L1^high^ tumor explants + CD4^+^ T cells was higher than that of the group of IL-21^low^/PD-L1^low^ tumor explants + CD4^+^ T cells (38.8 ± 8.5% *vs.* 25.8 ± 2.4%, respectively, P < 0.05) (**Figures 5A, B**). The anti-PD-1 antibody showed an inhibitory effect on Treg generation when the CD4^+^ T cells were cultured with the IL-21^high^/PD-L1^high^ tumor explants (31.3 ± 5.0% *vs.* 38.8 ± 8.5%, P < 0.05). In addition, the combination of IL-21 and the anti-PD-1 antibody had a stronger inhibitory effect on Treg generation than the anti-PD-1 antibody alone (26.6 ± 4.7% *vs.* 31.3 ± 5.0%, respectively, P < 0.05), whereas blocking IL-21 alone had no significant inhibitory effect on Treg generation (38.1 ± 7.1% *vs.* 38.8 ± 8.5%, P > 0.05). Conversely, the Treg generation induced by the IL-21^low^/PD-L1^low^ tumor explants failed to be reversed by neutralizing IL-21 (24.4 ± 3.7% *vs.* 25.8 ± 2.4%, P > 0.05), PD-1 (22.8 ± 2.4% *vs.* 25.8 ± 2.4%, P > 0.05), or both IL-21 and PD-1 (22.5 ± 3.1% *vs.* 25.8 ± 2.4%, P > 0.05), indicating that Treg generation in the IL-21^high^/PD-L1^high^ tumor microenvironment may be regulated by mechanisms distinct from those in the IL-21^low^/PD-L1^low^ tumor microenvironment (**Figures 5A, B**). Treg generation induced by IL-21^high^/PD-L1^high^ tumor explant was further validated by cocultured with TAA-specific T cells (both CD4 and CD8) (**Supplementary Figures 4**, **5**). Using intracellular staining, the cytokine-producing ability of responder CD4^+^ T cells and CD8^+^ T cells was assessed. Our results showed that the stimulated responder CD4^+^ and CD8^+^ T cells exhibited strong proliferation in both, high IFN-γ, IL-2 levels in responder CD4^+^ T cells, high GranzymeB and Perforin levels in responder CD8^+^ T cells. Adding the induced Treg by tumor explants with or without anti-PD-1 or/and anti-IL-21 treatment strongly inhibited the proliferation of responder T cells (both CD4 and CD8) (**Supplementary Figures 4A**, **5A**), reduced the IFN-γ, IL-2 levels of CD4^+^ T cells (**Supplementary Figures 4B, D**), and cytotoxicity, GranzymeB, Perforin levels of CD8^+^ T cells (**Supplementary Figures 5B, E**). However, no significant differences were observed between tumor explant with no antibody, with anti-PD-1, with anti-IL-21 group and with the combination anti-PD-1 and anti-IL-21. (**Supplementary Figures 4D, 5E**).There data suggested the Tregs induced by tumor explant with the treatment of anti-PD-1 or/and anti-IL-21 are expectedly suppressive as the Tregs induced by tumor explant alone.

The above data suggested that IL-21 neutralization may enhance the effect of PD-1-targeted tumor immunotherapy in only HNSCC patients with high expression of both IL-21 and PD-L1.

### Simultaneous High Expression of IL-21 and PD-L1 Is Associated With More Treg Infiltrates and Worse Survival

**Figure 6:**
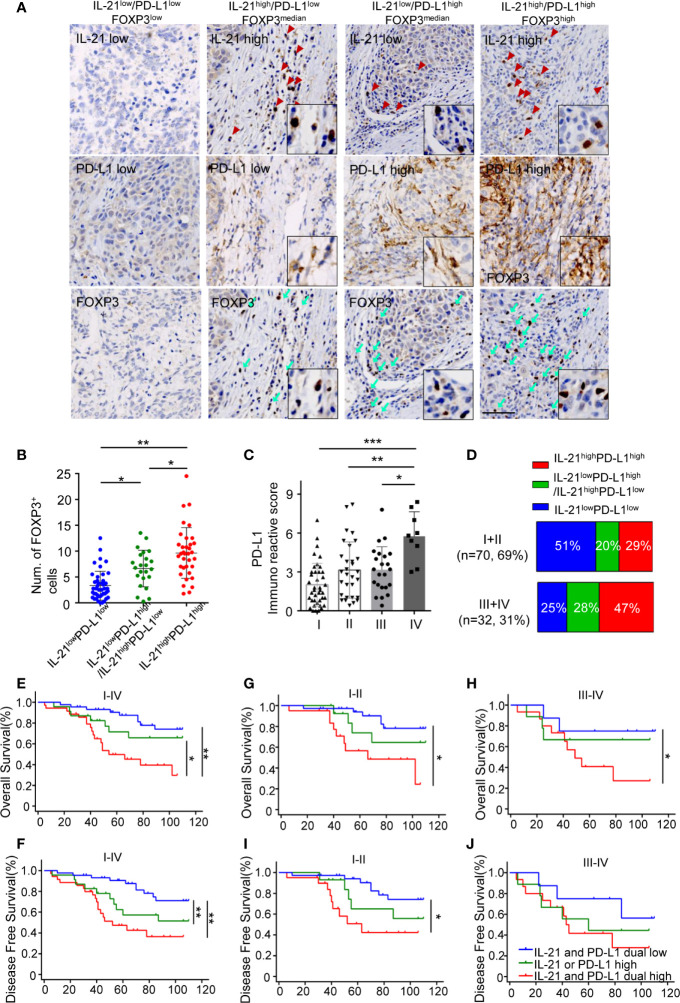
Simultaneous high expression of IL-21 and PD-L1 was associated with more Treg infiltrates and worse survival. **(A)** Representative images of IHC staining showed IL-21, B7-H1 and FOXP3 in serial sections. Scale bar represent 100μm, magnification, 200×. **(B)** Quantitative histogram showed that tumors with simultaneous high expression of IL-21 and B7-H1 have more FOXP3^+^ cells than those with simultaneous low expression of IL-21 and PD-L1 and those with high expression of either IL-21 or PD-L1. **(C)** B7-H1 expression in tumors with each of the stage I, II, III and IV, *P < 0.05. **P < 0.01, ***P < 0.001, by One way ANOVA **(D)** Comparison of the percentage of PD-L1 and/or IL-21 expression with stage I and II compared to those with stage III and IV. **(E–J)** Simultaneous high expression of IL-21 and B7-H1 predicted worse overall and disease-free survival in patients at stages I–IV **(E, F)**, stages I and II only **(G, I)**, or stages III and IV only **(H, J)** with HNSCC. (n (IL-21 and B7-H1 dual low expression) = 44, n (IL-21 or B7-H1 high expression) = 23, n (IL-21 and B7-H1 dual low expression) = 35, *P < 0.05. **P < 0.01). Number at risk and hazard ratio for survival plots has been provides in supplementary table section.

Given that IL-21 plays a positive role in PD-L1-induced Treg generation, we next investigated the relationships between the expression patterns of IL-21, PD-L1, and FOXP3 in tumor specimens. Our results showed that the tumors with high expression of IL-21 or PD-L1 had more FOXP3^+^ cells than those with low expression of both IL-21 and PD-L1 (6.8 ± 3.6 *vs.* 3.2 ± 2.7 counts per field, respectively, P < 0.05) (**Figures 6A, B**). Furthermore, the tumors with high expression of both IL-21 and PD-L1 had more FOXP3^+^ cells (9.6 ± 4.9 counts per field) than the tumors with low expression of both IL-21 and PD-L1 (3.2 ± 2.7 counts per field, P < 0.05) or the tumors with high expression of either IL-21 or PD-L1 (6.8 ± 3.6 counts per field, P < 0.01) (**Figures 6A, B**). Moreover, in the tumor stage analysis, the PD-L1 expression score in patients with stage IV disease was considerably higher than that in those with stage I, II or III disease (5.8 ± 1.9 *vs.* 2.0 ± 1.6, P <0.001, *vs.* 3.1 ± 2.2, P < 0.01, *vs.* 3.2 ± 1.8, P <0.05, respectively), (**Figure 6C**). Fifteen cases (47%) with simultaneous high expression of IL-21 and PD-L1 were observed among the stage III or IV samples, and 20 cases (29%) were observed among the stage I or II samples, while simultaneous low expression of IL-21 and PD-L1 was observed in 8 cases (25%) among the stage III or IV samples and in 36 cases (51%) among the stage I or II samples. Moreover, high expression of either PD-L1 or IL-21 was observed in 9 cases (28%) among the stage III or IV samples, and 14 cases (20%) were observed among the stage I or II samples (**Figure 6D**). We finally evaluated whether the simultaneous high expression of IL-21 and PD-L1 correlated with the clinical prognosis of HNSCC patients. As expected, the patients with simultaneous high expression of IL-21 and PD-L1 had worse overall and disease-free survival than those with simultaneous low expression of PD-L1 and IL-21 (OS: P < 0.01, DFS: P < 0.01), worse overall survival than high expression of either PD-L1 or IL-21 (P < 0.05, DFS not significant) (**Figures 6E, F**). Overall survival was still significantly different between IL-21^high^/PD-L1^high^ and IL-21^low^/PD-L1^low^ group at stages I + II (P < 0.05) and III + IV (P < 0.05), respectively (**Figures 6G, H**). Disease-free survival was significantly different between IL-21^high^/PD-L1^high^ and IL-21^low^/PD-L1^low^ group at stages I + II (P < 0.05), but not stages III + IV (P > 0.05) (**Figures 6I, J**). Cox regression analysis revealed that the simultaneous high expression of PD-L1 and IL-21 was an independent prognostic marker for overall and disease-free survival in HNSCC patients (**Table 3**).

## Discussion

Substantial evidence shows that immune cells are habitually recruited into the tumor microenvironment, where they create inflammatory responses and play a critical role in the establishment of the immunosuppressive milieu (23, 24). The present study demonstrated that the tumor microenvironment inflammatory factor IL-21 may promote PD-L1-induced, Treg-mediated tumor immune escape in a PD-1-dependent manner. These findings provide evidence that the tumor microenvironment inflammation represented by IL-21 plays an important role in inhibiting antitumor immune responses *via* Treg generation.

To date, few studies have shown that IL-21-associated inflammation exists in human solid tumors, and the immune significance of IL-21 in the tumor microenvironment remains controversial. Some studies have revealed that IL-21 may enhance the cytotoxic activity of CD8^+^ T cells and natural killer (NK) cells, suggesting that IL-21 functions as an antitumor agent (25, 26), while other studies have reported the immunosuppressive significance of IL-21 in promoting tumor-associated macrophage polarization and the expression of IL-10 by cytotoxic cells and B cells (12, 14, 15). We speculated that these inconsistencies could be attributed to the different tumor microenvironment contexts, IL-21 has been proposed acting in a context-dependent manner (27), therefore while presence of IL-21 provide a valid explanation for the activation of T or B cells in tumor tissue, a variety of cofactors e.g., PD-L1, CTLA-4 and tumor-derived factors may influence IL-21 actions. Moreover, local presence of IL-21 within tumor microenvironment is likely to interact with a variety of cell types or environmental factors than its systemic occurrence, for PD-1 ligation was observed to skew TCR repertories (28). It is thus possible that IL-21 selectively support a local immunosuppressive environment, whereas systemic application of IL-21 activates cytotoxic T or NK cells with anti-tumor activity at tumor-distant sites. Our hypothesis is supported by the proposal that tumor immune evasion is selectively modulated in local tumor microenvironment (22). Therefore, it likely depends on the status and type of immune infiltrates and the presence of environmental cofactors such as PD-L1 whether anti-tumor immune response is suppressed or supported by IL-21 in tumor microenvironment.

Recent studies reported the discrepant transcriptional and functional properties between tumor infiltrating Tregs, tissue resident Tregs and peripheral blood Tregs, indicating that tumor microenvironment may play an essential role in the regulation of the phenotype and immunosuppressive functionality of Tregs (29, 30). We recently showed that HNSCC-associated inflammation can augment the tumor microenvironment Treg population, which implied the inflammatory significance of Treg generation (10). However, the specific relationship between TAI and Treg generation is currently unclear. The present study aimed to evaluate the mechanisms by which IL-21-mediated inflammation regulates the generation and function of Tregs in the tumor microenvironment.

To our knowledge, this is the first study to propose significant crosstalk between IL-21 and PD-L1 in the generation of the tumor microenvironment Tregs that are responsible for the enhanced suppression of TAA-specific T cell proliferation. In detail, we first examined the effect of IL-21 alone on Treg generation, and the results showed that IL-21 inhibited Treg generation, which was inconsistent with our immunohistochemical data that showed that Treg infiltrates positively correlated with the counts of IL-21-positive cells. The above results implied that other tumor microenvironment elements may be involved in the regulation of Treg generation. As PD-L1/PD-1 signaling has recently been shown to modulate the Treg homeostasis and facilitate tumor immune tolerance (18–21), we hypothesized that PD-L1/PD-1 signaling may be involved in the process of Treg generation regulated by IL-21. Hence, we next added PD-L1 to IL-21-treated CD4^+^ T cells and found that IL-21 significantly promoted Treg generation in the presence of PD-L1. The potential mechanism is that IL-21 can induce the expression of PD-1 for PD-L1/PD-1 signaling, which is responsible for Treg generation. The mechanism of the upregulation of PD-1 expression induced by IL-21 might be a TCR-triggered calcineurin signaling cascade that leads to the activation of the transcription factors NFATc1 and STAT family proteins (31, 32). Moreover, although a recent study reported that cancer cells produced TGF-β1 in tumor supernatants and when CD4^+^ T cells were cultured in tumor supernatants, FOXP3^+^ Tregs generation was promoted. While IL-21 was able to inhibit cancer cell-mediated FOXP3 induction in the context of tumor supernatants (33), which was contrary to our study. We believe that this inconsistency may be attributed to that study ignoring the involvement of the tumor microenvironment, and IL-21 may exert double sword effects on Tregs at the context of other immunosuppressive component derived from tumor microenvironment such as PD-L1.

To validate the synergistic effect of IL-21 and PD-L1 on Treg generation in the tumor microenvironment, we used tumor explants freshly isolated from cancer patients to induce Treg generation from CD4^+^ T cells and to test whether neutralizing IL-21 and PD-1 was capable of blocking Treg generation. Our results showed that compared with either treatment alone, IL-21 neutralization combined with an anti-PD-1 neutralizing antibody could effectively inhibit IL-21^high^/PD-L1^high^ tumor explant-induced Treg generation, whereas this effect was not observed with IL-21^low^/PD-L1^low^ tumor explant-induced Treg generation, indicating that other mechanisms might be involved in Treg generation in tumor microenvironments with low expression of IL-21 and PD-L1. The potential mechanisms that may be involved in Treg generation involve other mediators, such as TGF-β, IL-10, and IDO, and interactions with tolerogenic DCs or stromal cells in the tumor milieu (34–38). The above clinical data suggest that the IL-21 neutralization strategy may represent a promising immunotherapeutic approach that may enhance PD-1 blockade-based tumor immunotherapy by targeting Treg-mediated immune evasion in only patients with high expression of IL-21 and PD-L1.

To expand the understanding of the correlations between IL-21, PD-L1, and Tregs in the tumor microenvironment and their prediction of disease prognosis, we detected the expression of IL-21, PD-L1, and FOXP3 in 102 newly diagnosed HNSCC patients. Our results showed that enriched IL-21^+^ cells and PD-L1 expression were positively associated with FOXP3^+^ cell infiltration and that high expression of IL-21 and PD-L1 correlated with advanced tumor stage and poor overall and disease-free survival in patients with HNSCC.

In conclusion, our results suggest that increased IL-21-associated inflammation in the tumor milieu may favor Treg-mediated immune evasion through the PD-L1/PD-1 pathway and that the IL-21 neutralization strategy may enhance PD-1 blockade-based tumor immunotherapy by targeting Treg-mediated immune evasion in patients with high expression of IL-21 and PD-L1. Understanding the mechanisms of the interactions between Tregs and tumor microenvironment inflammation may be beneficial for optimizing Treg-targeted antitumor strategies.

## Data Availability Statement

The raw data supporting the conclusions of this article will be made available by the authors, without undue reservation.

## Ethics Statement

The studies involving human participants were reviewed and approved by Ethics Committee of the First Affiliated Hospital of Sun Yat-sen University. The patients/participants provided their written informed consent to participate in this study.

## Author Contributions

Conception and design: WS and WW. Acquisition of data: YZ, ZZ, YW, LC, KL, HL, YX, RM, and FW. Analysis and interpretation of data: WS, YHW, WL, and FJ. Writing, review, and/or revision of the manuscript: YZ, ZZ, WS, and WW. Study supervision: WS and WW. All authors contributed to the article and approved the submitted version.

## Funding

This work was supported by grants from the Natural Science Foundation of Guangdong Province (2018B030312008, 2016A030310153, 2014A030313031, 2016A030313257, 2017A030310362, and 2018A030313667), the Science and Technology Planning Project of Guangdong Province (2014A020212141, 201704020098), the Natural Science Foundation of China (81870696, 81602365, 81670902, 81470674 and 81972527), the Medical Scientific Research Foundation of Guangdong Province (A2017216), the Science and Technology Program of Guangzhou (201704020098), and the Guangzhou Key Laboratory of Otorhinolaryngology Head and Neck Surgery (201605030003).

## Conflict of Interest

The authors declare that the research was conducted in the absence of any commercial or financial relationships that could be construed as a potential conflict of interest.

## Supplementary Material

Supplementary Figure 1 Gating strategy of flow cytometric plot for tumor infiltrating lymphocytes.

Supplementary Figure 2
**(A)** Representative flow cytometric histograms of the expression of IL-21R on Treg cells and conventional T cells (CD45+CD3+CD4+CD8-CD25-FOXP3-) in tumor are shown; **(B)** The frequencies of IL-21R+ Treg cells were significantly higher compared with conventional T cells, n=5, *P < 0.05.

Supplementary Figure 3 Proliferation of CD8+ T responder cells were assessed after 72h by flow cytometry. **(A, B)** Induced Tregs were cocultured with CFSE labeled TAA- specific CD8+ T responder cells at ratios of 1:4 and in the presence of anti-CD3/anti-CD28 antibodies. n=5, *P < 0.05.

Supplementary Figure 4 Induced Treg cocultured with TAA-specific CD4+ T cells. Tregs induced by IL-21high/PD-L1high tumor explants were cocultured with CD4+ T responder cells at ratios of 1:4 and in the presence of anti-CD3/anti-CD28 antibodies. **(A)** Proliferation of CFSE labeled TAA CD4+ T responder cells were assessed after 72h by flow cytometry. Production of IFN-γ **(B)**, IL-2 **(C)** in cocultured CD4+ T responder cells was detected by intracellular staining. **(D)** Quantification of proliferation and percentage of IFN-γ, IL-2 of CD4+ T responder cells. n=5, n.s., not significant.

Supplementary Figure 5 Induced Treg cocultured with TAA-specific CD8+ T cells Tregs induced by IL-21high/PD-L1high tumor explants were cocultured with CD8+ T responder cells at ratios of 1:4 and in the presence of anti-CD3/anti-CD28 antibodies. **(A)** Proliferation of CFSE labeled TAA CD8+ T responder cells were assessed after 72h by flow cytometry. **(B)** TAA-specific cytotoxicity was determined by flow cytometry. Production of GranzymeB **(C)**, Perforin **(D)** in cocultured CD8+ T responder cells was detected by intracellular staining. **(E)** Quantification of proliferation, TAA-specific cytotoxicity and percentage of GranzymeB, Perforin of CD8+ T responder cells. n=5, n.s., not significant.
