# Supplementary figures and images for: IL-21 Is an Accomplice of PD-L1 in the Induction of PD-1-Dependent Treg Generation in Head and Neck Cancer
Source: Front Oncol. 2021 May 5;11:648293. doi: 10.3389/fonc.2021.648293 (PMC8131831; doi:10.3389/fonc.2021.648293)

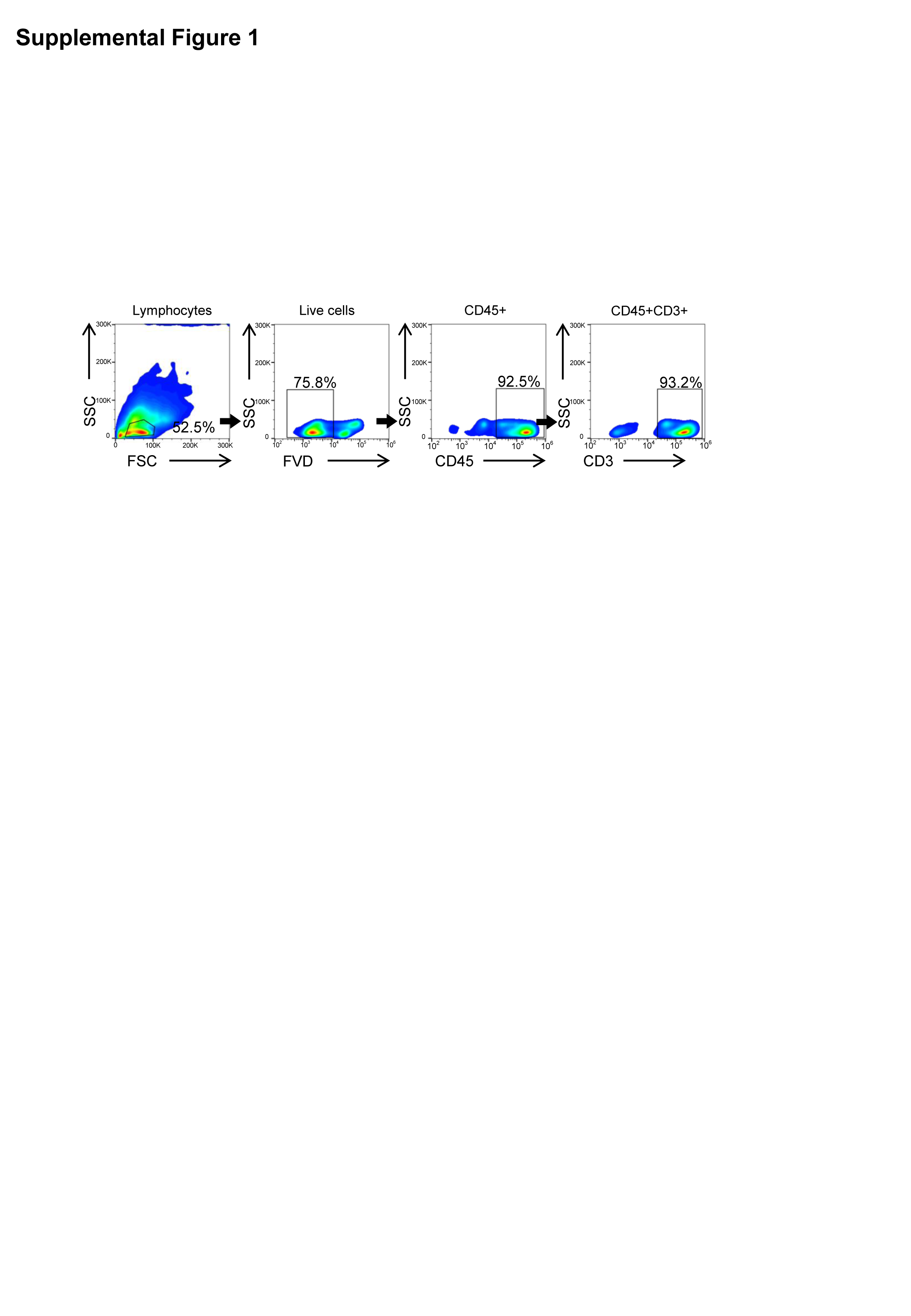

Supplement: Supplementary Figure 1 — Gating strategy of flow cytometric plot for tumor infiltrating lymphocytes. [file Image_1.tif]

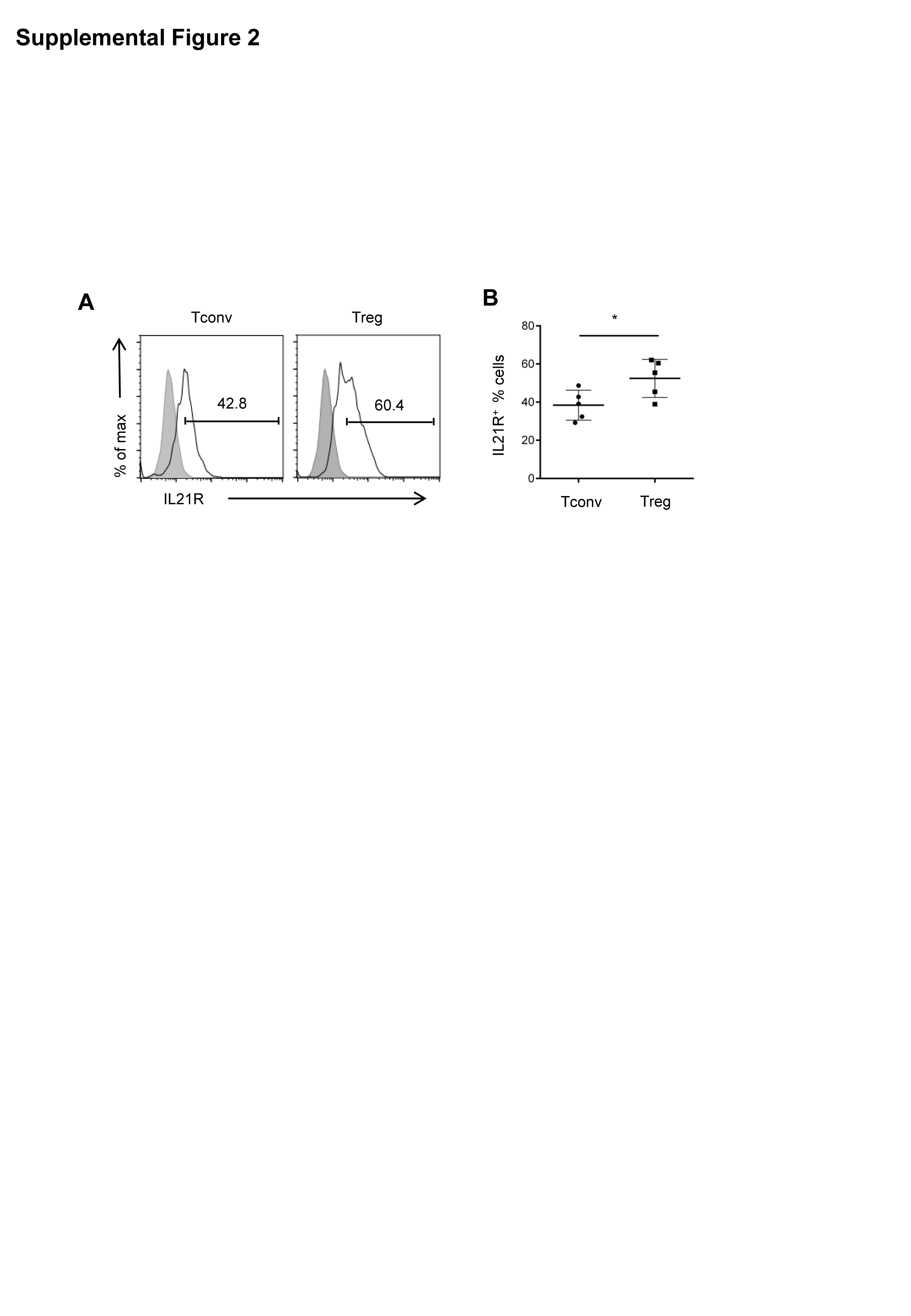

Supplement: Supplementary Figure 2 — (A) Representative flow cytometric histograms of the expression of IL-21R on Treg cells and conventional T cells (CD45+CD3+CD4+CD8-CD25-FOXP3-) in tumor are shown; (B) The frequencies of IL-21R+ Treg cells were significantly higher compared with conventional T cells, n=5, *P < 0.05. [file Image_2.tif]

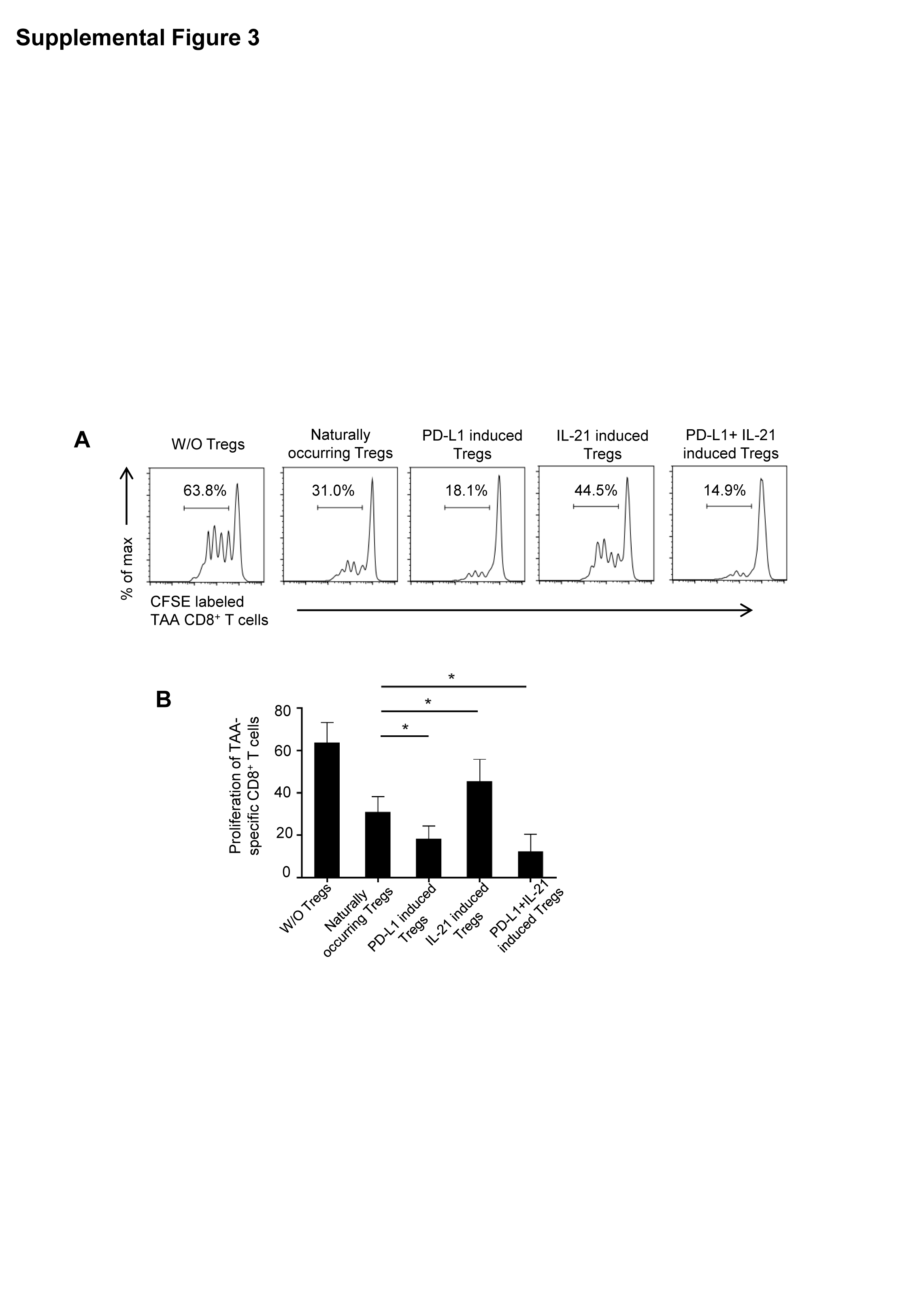

Supplement: Supplementary Figure 3 — Proliferation of CD8+ T responder cells were assessed after 72h by flow cytometry. (A, B) Induced Tregs were cocultured with CFSE labeled TAA- specific CD8+ T responder cells at ratios of 1:4 and in the presence of anti-CD3/anti-CD28 antibodies. n=5, *P < 0.05. [file Image_3.tif]

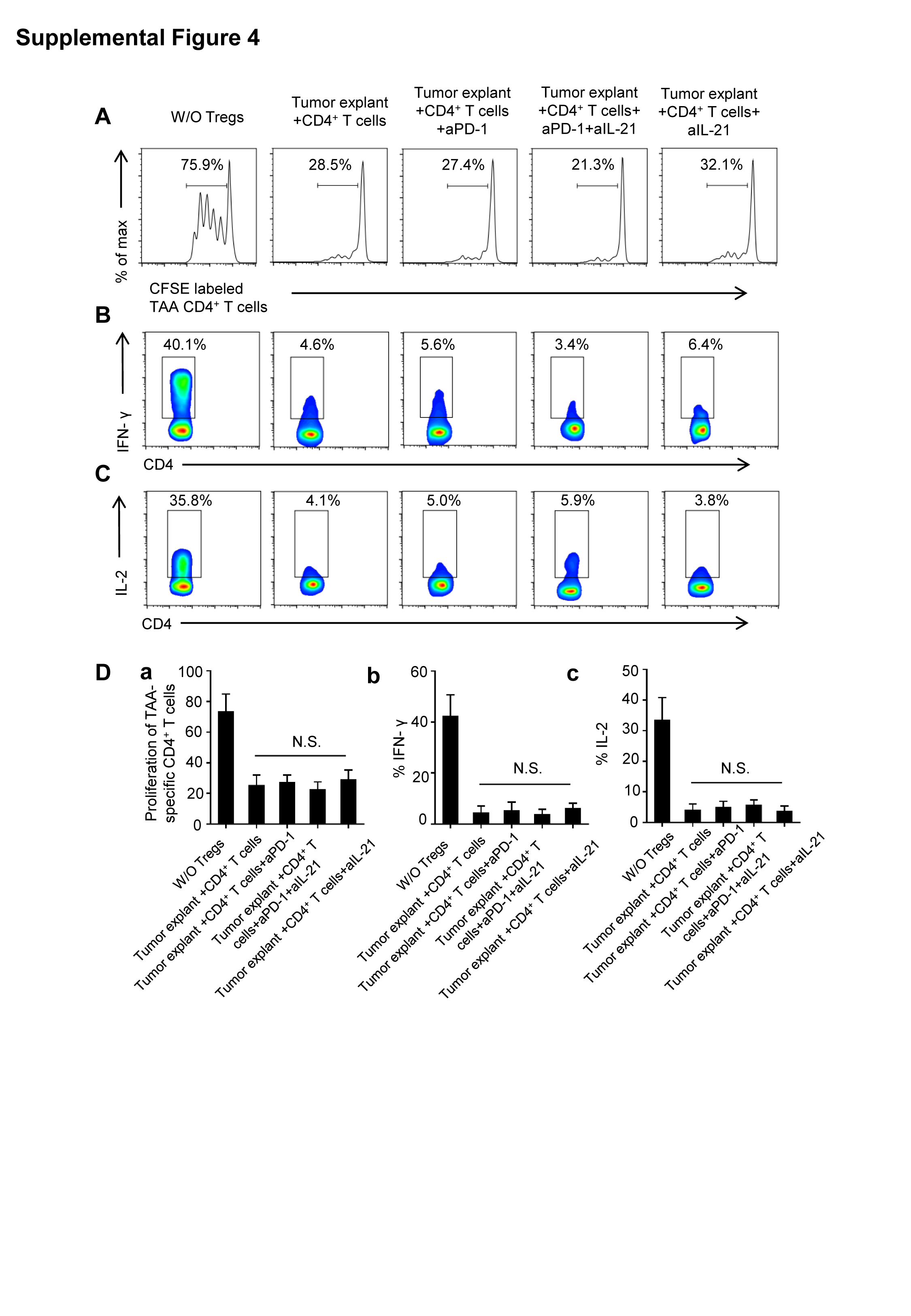

Supplement: Supplementary Figure 4 — Induced Treg cocultured with TAA-specific CD4+ T cells. Tregs induced by IL-21high/PD-L1high tumor explants were cocultured with CD4+ T responder cells at ratios of 1:4 and in the presence of anti-CD3/anti-CD28 antibodies. (A) Proliferation of CFSE labeled TAA CD4+ T responder cells were assessed after 72h by flow cytometry. Production of IFN-γ (B), IL-2 (C) in cocultured CD4+ T responder cells was detected by intracellular staining. (D) Quantification of proliferation and percentage of IFN-γ, IL-2 of CD4+ T responder cells. n=5, n.s., not significant. [file Image_4.tif]

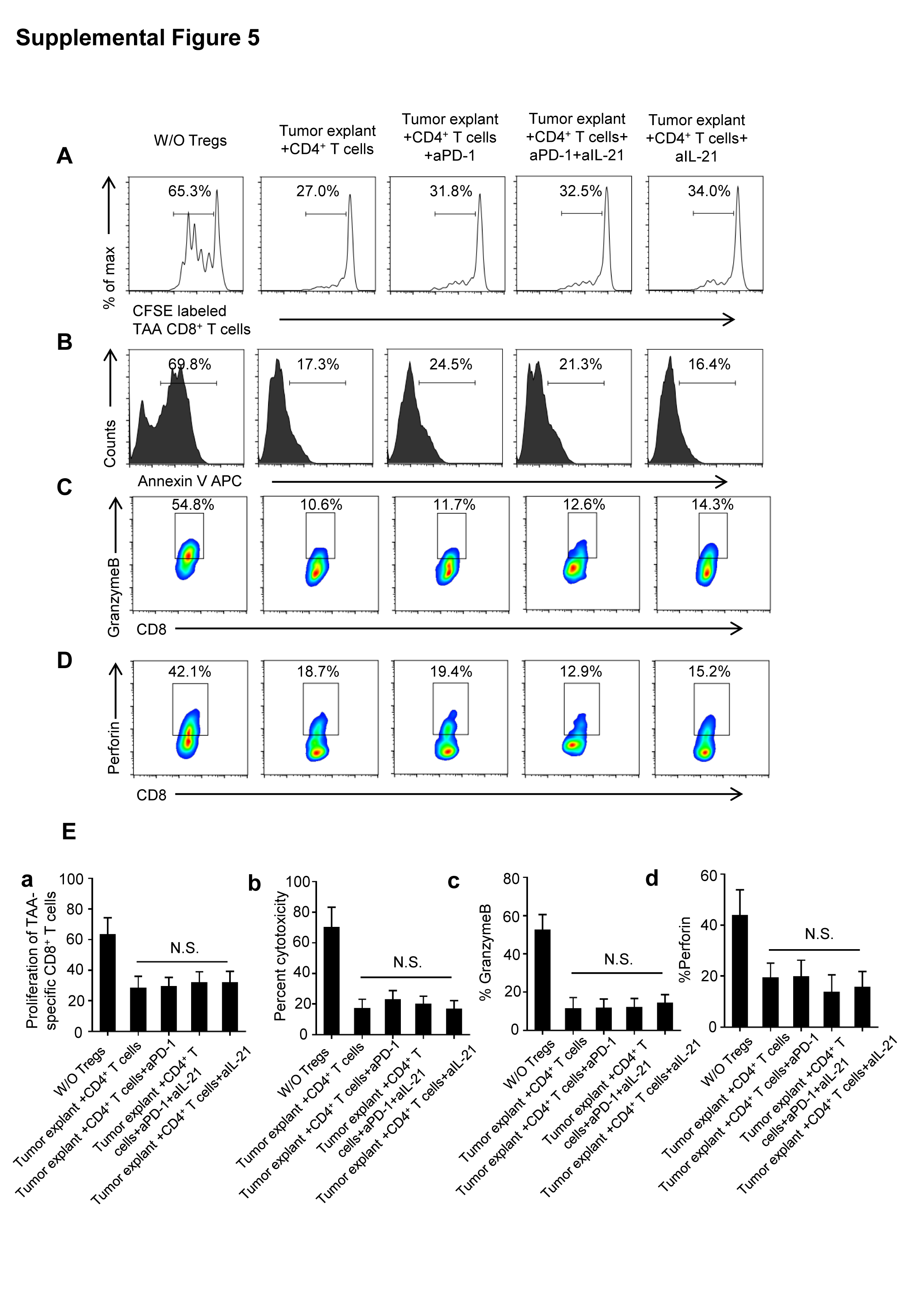

Supplement: Supplementary Figure 5 — Induced Treg cocultured with TAA-specific CD8+ T cells Tregs induced by IL-21high/PD-L1high tumor explants were cocultured with CD8+ T responder cells at ratios of 1:4 and in the presence of anti-CD3/anti-CD28 antibodies. (A) Proliferation of CFSE labeled TAA CD8+ T responder cells were assessed after 72h by flow cytometry. (B) TAA-specific cytotoxicity was determined by flow cytometry. Production of GranzymeB (C), Perforin (D) in cocultured CD8+ T responder cells was detected by intracellular staining. (E) Quantification of proliferation, TAA-specific cytotoxicity and percentage of GranzymeB, Perforin of CD8+ T responder cells. n=5, n.s., not significant. [file Image_5.tif]
