# Supplementary material for: IL-21 Is an Accomplice of PD-L1 in the Induction of PD-1-Dependent Treg Generation in Head and Neck Cancer
Source: Front Oncol. 2021 May 5;11:648293. doi: 10.3389/fonc.2021.648293 (PMC8131831; doi:10.3389/fonc.2021.648293)
Supplement: Supplementary file 6 [file DataSheet_1.docx]

**Supplementary table**

**Number at risk and hazard ratio for survival plots in Fig 2C-2D, 6E-6J**

**Fig 2C**

| Number at risk | 0 | 20 | 40 | 60 | 80 | 100 | HR(95%CI) |
| --- | --- | --- | --- | --- | --- | --- | --- |
| Low IL-21 | 58 | 58 | 50 | 37 | 27 | 24 | 0.45 (0.21-0.84) |
| High IL-21 | 44 | 42 | 32 | 20 | 16 | 13 |  |

**Fig 2D**

| Number at risk | 0 | 20 | 40 | 60 | 80 | 100 | HR(95%CI) |
| --- | --- | --- | --- | --- | --- | --- | --- |
| Low IL-21 | 58 | 58 | 50 | 36 | 27 | 25 | 0.47 ( 0.23-0.9) |
| High IL-21 | 44 | 40 | 29 | 20 | 16 | 11 |  |

**Fig 6E**

| Number at risk | 0 | 20 | 40 | 60 | 80 | 100 | HR(95%CI) |
| --- | --- | --- | --- | --- | --- | --- | --- |
| IL-21 and PD-L1 dual low | 44 | 44 | 40 | 32 | 23 | 21 | 0.25(0.09-0.46) |
| Il-21 or PD-L1 high  IL-21 and PD-L1 dual high | 23 | 23 | 19 | 14 | 13 | 12 | 0.45(0.20-0.93) |
|  | 35 | 34 | 24 | 12 | 8 | 5 | Reference |

**Fig 6F**

| Number at risk | 0 | 20 | 40 | 60 | 80 | 100 | HR(95%CI) |
| --- | --- | --- | --- | --- | --- | --- | --- |
| IL-21 and PD-L1 dual low | 44 | 44 | 40 | 32 | 23 | 20 | 0.27(0.10-0.49) |
| Il-21 or PD-L1 high  IL-21 and PD-L1 dual high | 23 | 23 | 18 | 12 | 12 | 10 | 0.62(0.29-1.3) |
|  | 35 | 32 | 22 | 11 | 7 | 4 | Reference |

**Fig 6G**

| Number at risk | 0 | 20 | 40 | 60 | 80 | 100 | HR(95%CI) |
| --- | --- | --- | --- | --- | --- | --- | --- |
| IL-21 and PD-L1 dual low | 36 | 36 | 34 | 26 | 19 | 18 | 0.22(0.07-0.45) |
| Il-21 or PD-L1 high  IL-21 and PD-L1 dual high | 14 | 14 | 13 | 9 | 8 | 8 | 0.40 (0.13-1.19) |
|  | 20 | 20 | 14 | 8 | 6 | 3 | Reference |

**Fig 6H**

| Number at risk | 0 | 20 | 40 | 60 | 80 | 100 | HR(95%CI) |
| --- | --- | --- | --- | --- | --- | --- | --- |
| IL-21 and PD-L1 dual low | 8 | 8 | 7 | 7 | 6 | 4 | 0.28(0.10-0.87) |
| Il-21 or PD-L1 high  IL-21 and PD-L1 dual high | 9 | 9 | 7 | 6 | 6 | 5 | 0.47(0.16-1.39) |
|  | 15 | 15 | 10 | 5 | 3 | 3 | Reference |

**Fig 6I**

| Number at risk | 0 | 20 | 40 | 60 | 80 | 100 | HR(95%CI) |
| --- | --- | --- | --- | --- | --- | --- | --- |
| IL-21 and PD-L1 dual low | 36 | 36 | 34 | 26 | 19 | 18 | 0.22(0.04-0.46) |
| Il-21 or PD-L1 high  IL-21 and PD-L1 dual high | 14 | 14 | 13 | 8 | 8 | 7 | 0.52(0.18-1.50) |
|  | 20 | 20 | 13 | 7 | 5 | 2 | Reference |

**Fig 6J**

| Number at risk | 0 | 20 | 40 | 60 | 80 | 100 | HR(95%CI) |
| --- | --- | --- | --- | --- | --- | --- | --- |
| IL-21 and PD-L1 dual low | 8 | 8 | 7 | 7 | 5 | 4 | 0.42(0.14-1.42) |
| Il-21 or PD-L1 high  IL-21 and PD-L1 dual high | 9 | 9 | 6 | 5 | 5 | 4 | 0.79(0.27-2.35) |
|  | 15 | 13 | 9 | 5 | 3 | 3 | Reference |
